# Use of artificial intelligence in sports medicine: a report of 5 fictional cases

**DOI:** 10.1186/s13102-021-00243-x

**Published:** 2021-02-16

**Authors:** Lia Rigamonti, Katharina Estel, Tobias Gehlen, Bernd Wolfarth, James B. Lawrence, David A. Back

**Affiliations:** 1 Center of Sport Medicine, Department Sport and Health Science, University of Potsdam, University Outpatient Clinic, Am Neuen Palais 10, 14469 Potsdam, Germany; 2 Clinic of Traumatology and Orthopedics, Bundeswehr Hospital Berlin, Berlin, Germany; 3 Center for Musculoskeletal Surgery, Charité University Medicine Berlin, Berlin, Germany; 4 Department of Sports Medicine, Humboldt University and Charité University Medicine Berlin, Berlin, Germany; 5 Department of Health and Physical Education, Mercer County Community College, West Windsor, NJ USA; 6 Dieter Scheffner Center for Medical Teaching and Educational Research, Charité University Medicine Berlin, Berlin, Germany

**Keywords:** Artificial intelligence, App, Sport medicine, Orthopedics, Pathologies

## Abstract

**Background:**

Artificial intelligence (AI) is one of the most promising areas in medicine with many possibilities for improving health and wellness. Already today, diagnostic decision support systems may help patients to estimate the severity of their complaints. This fictional case study aimed to test the diagnostic potential of an AI algorithm for common sports injuries and pathologies.

**Methods:**

Based on a literature review and clinical expert experience, five fictional “common” cases of acute, and subacute injuries or chronic sport-related pathologies were created: Concussion, ankle sprain, muscle pain, chronic knee instability (after ACL rupture) and tennis elbow. The symptoms of these cases were entered into a freely available chatbot-guided AI app and its diagnoses were compared to the pre-defined injuries and pathologies.

**Results:**

A mean of 25–36 questions were asked by the app per patient, with optional explanations of certain questions or illustrative photos on demand. It was stressed, that the symptom analysis would not replace a doctor’s consultation. A 23-yr-old male patient case with a mild concussion was correctly diagnosed. An ankle sprain of a 27-yr-old female without ligament or bony lesions was also detected and an ER visit was suggested. Muscle pain in the thigh of a 19-yr-old male was correctly diagnosed. In the case of a 26-yr-old male with chronic ACL instability, the algorithm did not sufficiently cover the chronic aspect of the pathology, but the given recommendation of seeing a doctor would have helped the patient. Finally, the condition of the chronic epicondylitis in a 41-yr-old male was correctly detected.

**Conclusions:**

All chosen injuries and pathologies were either correctly diagnosed or at least tagged with the right advice of when it is urgent for seeking a medical specialist. However, the quality of AI-based results could presumably depend on the data-driven experience of these programs as well as on the understanding of their users. Further studies should compare existing AI programs and their diagnostic accuracy for medical injuries and pathologies.

**Supplementary Information:**

The online version contains supplementary material available at 10.1186/s13102-021-00243-x.

## Background

The current age of digital health offers a wide range of possibilities for improving medical care. Various technologies, such as telemedicine with video consultation or mobile health (mHealth) applications can already be considered established [1, 2], and the potentials of digitalization among sports, exercise, and orthopedics have been previously reviewed [3, 4]. However, the use of self-learning algorithms or artificial intelligence (AI) is still in the beginning stages of development. An early study has shown that AI-controlled algorithms could exceed the accuracy of predicting cardiovascular risk compared to guidelines of medical societies [5]. While areas such as robot-guided surgical interventions are still in a rather experimental state [6], analysis of radiological or dermatologic images have already reached milestones by achieving similar or even superior results when compared to humans [7, 8].

Patients commonly google their symptoms to find the causes or identification of their complaints, which on the one hand can lead to patient empowerment [9], but on the other hand could also lead to safety risks due to inaccuracy of the search results [10] and may amplify emotional distress [11]. Here, the use of AI-based algorithms in a diagnostic decision support system (DDSS) can be seen as another potential of AI in medicine. Data has already shown that such a DDSS can help patients to estimate the severity of their complaints and can also be useful to support medical personnel [12, 13].

In the field of sports medicine, only a few applications of AI have been described so far [14]. However, there are an estimated 8.6 million sports-related injuries reported annually in the USA. Therefore, a high potential for a DDSS can be assumed [15] - especially in the large field of amateur sports, where team doctors or athletic trainers are typically not immediately on hand. Additionally, these DDSS may help aid on-duty doctors or athletic trainers to potentially increase their accuracy of identifying specific injuries (e.g. concussion severity) in the field. AI-algorithms might help streamline doctor or emergency department visits by providing reliable, big data and quality-based recommendations concerning the severity of patients’ complaints.

The present case study is intended as an orientation to estimate the potential of an AI-application (app), testing the ability of identification and recommendation of actions for some typical sports injuries and pathologies.

## Methods

### Five fictional cases

Basing on an informal literature search on pubmed.gov, and from a consensus among the authors, five common acute and subacute injuries, and chronic pathologies in the field of sports medicine were identified. These were: Concussion, ankle sprain, muscle pain in the thigh, chronic knee instability after anterior crucial ligament (ACL) rupture and tennis elbow. No real patient records where involved in this approach. The ethical principles of the declaration of Helsinki were followed [16]. Based on the literature and the clinical experience of the authors, 5 fictional patient case vignettes were created with fictional patient’s histories and fictional examination results and tested within the AI-app Ada (Ada Health, Berlin, Germany).

### Function of the chosen AI app

The AI-app selected for the present case report is available for free download in the usual app stores. Ada is a chatbot app that collects data from users by asking algorithm-generated questions while using natural language. The chatbot app will first start with six to seven standard questions (name, gender, age of the respective patients, smoking habits, diagnosed high blood pressure, or diabetes; in females: possibility of pregnancy). In the following, the main symptom or complaints shall be typed in and an answer can be chosen from given options. After this, a variable number of multiple choice or dichotomic questions is generated. It is possible to return to earlier answers and change them during the process. Also “I don’t know” is an option, if an answer cannot be given. To increase understanding, explanations of certain questions or terms can be elaborated on, or, in some cases, explanatory pictures of pathologies may be viewed. Before offering potential solutions for the complaints, the user is informed that these are not diagnoses and are not an exhaustive list, therefore, the user’s injury or pathology might not be included and that a medical doctor should be consulted. Finally, the most likely solutions are presented in a numerical order, along with recommendations of action, and explanations of backgrounds and treatment options.

## Results

In the following, the 5 fictional case vignettes are presented, together with the respective solutions proposed by the algorithm. Findings that were not covered by the initial vignettes, are pointed out. The general estimation, or “next steps”, of each case by the app is given. Also, notes on unclear/critical aspects of each case are made.

**Table:** 

**Case 1**	
**Patient basic data: Michael M., male, dob 01.01.1979 (41y/o), non-smoking, no treated hypertension, or diabetes.**	
**Symptom: Pain in the right lateral elbow region**	
**Fictional diagnosis: Lateral epicondylitis of the humerus (tennis elbow)**	
History: The patient has a normal desk job, but has been doing leisure sports for years. For 10 months now he no longer participates in his previous sport that primarily involved running and instead has started playing badminton with friends twice a week. During this time he has noticed pain in the right (dominant arm) lateral elbow and the nearby forearm muscles, especially in the days after training. This pain is intensified by activities with a firm grip (e.g. opening screw caps, carrying water boxes, etc). These specific complaints have never completely resided and instead have increased in the last few weeks, reaching a 5 of “moderate pain” intensity on a visual analogue scale (VAS), ranging from 1 no pain to 10 worst pain. The patient does not recall any past trauma to this region, and does not have any other complaints in other parts of the body. He has not yet seen a doctor for this reason, but because of the pain, which is now considered to be unpleasant, one day after a game he uses the AI app.	
Examination results: Pressure pain above the *lateral epicondyle* of the *humerus* and pressure pain in the proximal muscles of the forearm with tender muscles. No signs of inflammation such as redness, swelling/articular effusion, overheating, or any lasting disturbance of function in everyday life. Range of motion (ROM) is not restricted, peripheral circulation, motor function and sensitivity are intact.	
Not anticipated while creating the vignette (and filled in while using the app in this trial): No lumps under the skin on the elbow, no lumps under the skin on the forearm or hand, no muscle cramps in the arms and hands, no bruise on the arm, no reduced mobility of the fingers, no reduced mobility of the wrist.	
Number of symptom-related questions: 27	
General app estimation (“next steps”): People with symptoms similar to yours can usually manage their symptoms safely at home. You could also seek advice by visiting or contacting your local pharmacy. If your symptoms persist longer than expected, if they get worse, or if you notice new symptoms, you should consult a doctor for further assessment and advice.	
Suggested diagnoses: 1. Tennis elbow (can usually be managed at home): 7 out of 10 people with these symptoms had this condition (➔ suggested therapy: Cryotherapy, medication against pain and inflammation, and physical therapy) 2. Golfer’s elbow (can usually be managed at home): 2 out of 100 people with these symptoms had this condition. 3. Less likely causes: Injury due to chronic overuse of the forearm muscles (1 out of 1000 people with these symptoms had this condition).	
Note: no questions were asked about the specific side of the affected elbow (i.e. medial vs. lateral); it was not asked whether the complaints would also become stronger at rest or more so during activity (stated pain: moderate; theoretically: hardly at rest, stronger under stress).

**Table:** 

**Case 2**	
**Patient basic data: Sarah S., female, dob 01.01.1993, (27 y/o), not pregnant, non-smoking, no treated hypertension, or diabetes.**	
**Symptom: Pain in left Ankle (joint)**	
**Fictional diagnosis: Ankle sprain (distortion of the** ***anterior*** **fibulotalar** ***ligament*) left**	
History: 2 h earlier, the patient had twisted her left foot during volleyball causing supination trauma. With immediate onset of pain, she had stopped playing and limped to the side bench under careful axial load on her left leg. A teammate had given her an ice pack, so she had iced and slightly elevated the leg. Above the lateral ankle a larger swelling has formed, she doesn’t dare attempt full weightbearing anymore, because it is quite painful, which she subjectively reports as “strong” (VAS 8). Worried about a more serious injury, she now uses the app.	
Examination results: Swelling in the area of the anterior fibulotalar ligament, pressure pain at the anterior distal tip of the lateral malleolus, no pressure pain over syndesmosis or high fibula, muscles of the calf, medial malleolus, deltoid ligament or foot skeleton. Mobility limited due to pain, pronation still possible at approx. 5°, supination associated with pain, dorsal extension/plantar flexion approx. 10°, no wounds, no hematoma visible, peripheral circulation and sensitivity intact (motor function just restricted in the ankle joint).	
Not anticipated while creating the vignette: N/A	
Answers suspected not to be answered by the fictional patient: “Do you feel that your ankle is instable?”	
Number of symptom-related questions: 29	
General app estimation (“next steps”): People with symptoms similar to yours may require emergency care. If you think this is an emergency you should go to an emergency department without delay.	
Suggested diagnoses: 1. Sprained ankle without ligament rupture (seek emergency care): 3 out of 10 people with these symptoms had this condition 2. Lateral malleolus fracture (seek emergency care): 2 out of 10 people with these symptoms had this condition 3. Lateral ligament rupture of the ankle (seek emergency care): 2 out of 10 people with these symptoms had this condition 4. Ankle fracture, not further specified (seek emergency care): 1 out of 10 people with these symptoms had this condition	
Note: N/A

**Table:** 

**Case 3**	
**Patient basic data: Peter P., male, dob 01.01.2001, (19 y/o), non-smoking, no treated hypertension, or diabetes.**	
**Symptom: Pain in left upper thigh**	
**Fictional diagnosis: Delayed onset of muscle soreness**	
History: Yesterday the patient had gone on a long hike with friends in the mountains – for the first time in his life. The friends had walked about 15 km with each a 10 kg backpack on paved paths in hilly terrain. The patient sustained no trauma. He found the uphill climbs very tiring. His other sports activities have been limited to school sports and computer games. Movement is now hardly possible, he complains of strong pain (VAS 7) in the quadriceps muscles and the buttocks, and displays a strong limping gait. After a quiet night, he now woke up with the above-mentioned symptoms and decided to use the app since he is deeply concerned about this unknown condition.	
Examination results: No observed circumferential increase, pressure pain over the thigh muscles ventrally, not dorsally; pressure pain over the gluteal muscles; no discomfort in the lower leg or anywhere else in the body; only axial loading is possible. Active ROM in the hip and knee joint is limited due to the pain in the thigh; passive movement is possible with light stretching exercises. Peripheral circulation, motor function and sensitivity is intact, no wounds, no hematoma visible.	
Not anticipated while creating the vignette (and filled in while using the app in this trial): Feeling of heavy legs, no lumps under the skin of the thighs	
Answers suspected not to be answered by the fictional patient: N/A	
Number of symptom-related questions: 22	
General app estimation (“next steps”): People with symptoms similar to yours can usually manage their symptoms safely at home. You could also seek advice by visiting or contacting your local pharmacy. If your symptoms persist longer than expected, if they get worse, or if you notice new symptoms, you should consult a doctor for further assessment and advice.	
Suggested diagnoses: 1. Delayed-onset muscle soreness of the lower extremity (can usually be managed at home): 5 out of 10 people with these symptoms had this condition 2. Quadriceps strain (can usually be managed at home): 2 out of 10 people with these symptoms had this condition	
Note: N/A

**Table:** 

**Case 4**	
**Patient basic data: Thomas T, male, dob 01.01.1994, (26 y/o), smoking, no treated hypertension, or diabetes.**	
**Symptom: Swollen knee**	
**Fictional diagnosis: ACL rupture with chronic instability**	
History: The patient is an amateur soccer player. He does not engage in other sports, and besides soccer, he does not regularly run. Instead he primarily engages in resistance training of the upper body. About 5 months ago, he sustained a knee distortion trauma with pain during a soccer match shortly before the end of the season leading into the winter break. At that time, he also had swelling with pain in the knee, which improved after a few days of rest and sympathetic relief. He did not consult with a doctor, because the pain and swelling improved quickly. Having a good muscle status, he had no further complaints. For about 3 months, during winter break, he had paused playing soccer anyways and had not done any substitute running. He had no problems with his gait and was fine during his desk job and during leisure time. Only when he went down the stairs, he felt a slight instability in his knee and therefore preferred to hold on to the railing. But there were no real events of pain. Now, after resuming soccer, he sensed some instability during every weekend game, combined with pain in the knee joint (VAS 4–5), and swelling, which decreases after 3–4 days. After 5 days of symptomatic rest and almost no complaints, he talked with his friends about this annoying occurrence and how he was not sure of the cause. They suggested he use the app for getting some helpful information.	
Examination results: Normal gait, Zohlen sign negative, low effusion, no patella embracing pain, no overheating/redness, no pain on palpation over medial/lateral knee joint gap, the popliteal fossa or the tibial head, meniscus signs negative. Lachman test, anterior drawer test, pivot shift test positive; free ROM, peripheral circulation, motor function and sensitivity intact, thigh circumference (20 cm above the knee cap) ipsilateral reduced by 1 cm	
Not anticipated while creating the vignette (and filled in while using the app in this trial): No morning stiffness, no lumps under the skin behind the knee or over a joint, no shin pain, no calf pain	
Answers suspected not to be answered by the fictional patient: N/A	
Number of symptom-related questions: 30	
General app estimation (“next steps”): People with symptoms similar to yours may require emergency care. If you think this is an emergency you should go to an emergency department without delay.	
Suggested diagnoses: 1. Knee bursitis (seek medical advice): 3 out of 10 people with these symptoms had this condition. 2. Anterior cruciate ligament injury (seek emergency care): 1 in 10 people with these symptoms had this condition. 3. Patellar tendinitis (can usually be managed at home): 8 out of 100 people with these symptoms had this condition. 4. Popliteal cyst (seek medical advice): 7 out of 100 people with these symptoms had this condition. 5. Tractus iliotibialis syndrome (can usually be managed at home): 4 out of 100 people with these symptoms had this condition.	
Note: The patient would have found it difficult to answer many of the questions because the symptoms questioned were no longer present at the time of the examination.

**Table:** 

**Case 5**	
**Patient basic data: Marc C., male, dob 01.01.1997, (23 y/o), smoker, no treated hypertension, or diabetes.**	
**Symptom: Headache**	
**Fictional diagnosis: Mild concussion (I°)**	
History: During an amateur soccer game (summer, sunny, 26 °C), the patient jumped after the ball and bumped his head against the knee of an opponent player, 1 h ago. No unconsciousness, no vomiting. He notices slight dizziness, which would become worse when standing up or while walking. Leading symptom is a strong dull headache, especially in the area of impact on the back of the head. This area is also painful to the touch. Tilting the head forward intensifies the headache. This is accompanied by moderate nausea. Otherwise, however, the young patient is awake, actively talking and moving, oriented and responsive. No other symptoms reported. He had retreated to a cool room within the sports facility and had drunk moderate amounts of water. Since he still had complaints and was generally dazed, other players advised him to consult the app.	
Examination results: Patient awake, oriented and cooperative. Retrograde amnesia to the impact event itself; otherwise normal memory of the situation immediately before the trauma, the soccer game, and also the time after the trauma. Headache with painful pressure over the impact region at the back of the head, but no wounds or hematoma. While walking freely slight problems of balance were indicated, but no objective clear swaying, no nystagmus, no pain on pressure or other complaints in the facial region, no discharge from the ears. Visual acuity intact, no eye pain. Cervical and neck region freely movable without pain (also no complaints when moving against resistance).	
Not anticipated while creating the vignette (and filled in while using the app in this trial): No lumps under the skin on the scalp, no jerking movements of the whole body, no recent decrease in alcohol intake	
Answers suspected not to be answered by the fictional patient: N/A	
Number of symptom-related questions: 33	
General app estimation (“next steps”): People with symptoms similar to yours may require emergency care. If you think this is an emergency the safest thing to do is call an ambulance.	
Suggested diagnoses: 1. Concussion (seek medical advice): 5 out of 10 people with these symptoms had this condition. 2. Acute subdural hematoma (seek emergency care): 1 out of 10 people with these symptoms had this condition. 3. Whiplash (seek medical advice): 3 out of 100 people with these symptoms had this condition. 4. Acute intracranial epidural hematoma (seek emergency care): 2 out of 100 people with these symptoms had this condition. 5. Skull fracture (seek emergency care): 1 out of 100 people with these symptoms had this condition.	
Note: A worsening of the condition, e.g. a clouding with slowly progressive brain swelling, could not necessarily be detected with the single app use.	

## Discussion

Even with the rapid development and use of digitization in healthcare, there is still a very large potential in the field of artificial intelligence (AI) [17]. Among many other areas, diagnostic decision support systems (DDSS) seem to be particularly promising. Especially in the field of sports medicine, where acute injuries but also chronic pathologies are common not only in elite athletes but also in amateurs [15]. While some traumatic injuries and severe pathologies will require immediate involvement of medical personal, the use of a DDSS might help patients and medical professionals to better understand less clear complaints more effectively, resulting in faster and more proper treatment. To establish an initial impression of the potential of a DDSS, 5 different fictional sport injuries and pathologies were analyzed with an AI chatbot app in this case report.

Concerning the selected injuries and pathologies of this report, the authors felt these are fairly typical, and are widely occurring among a variety of different sports. In the case of “simple complaints” such as muscle pain or tennis elbow, the information provided by the app may help assist the patient to self-treat on their own. Although, it should be critically noted that the tennis elbow diagnosis could have been more precise if the app was more knowledgeable about orthopedic pathologies and questioned about the medial versus lateral side of the elbow. In the case of the ankle sprain, the recommendation to visit an emergency unit is to be considered satisfactory, since it may be hard to distinguish fractures from ligament injuries after such a trauma and therefore a professional medical examination (with possible x-ray) could provide decisive clarity [18]. Also, in the case with the concussion, the recommendation to consult a doctor is certainly correct [19]. Here, the biggest criticism is that the recommendation did not take time into consideration, which could cause a delay, resulting in a more dire outcome. Hence, a guideline-corresponding follow-up by AI algorithms after minutes to hours could be built into the app to enable a fast implementation from the recommendations [20]. Other studies have already shown significant differences in diagnostic capacities among different algorithms in the context of concussion [21], but data also suggested a great potential of AI diagnostic support as assisting tool to clinicians [22].

The app used in the present case report showed weaknesses in chronic ACL instability, since the reoccurring chronic character of the condition was not sufficiently captured by the apps selected questions and the suggested main diagnosis of a bursitis would rather not have gone along with a feeling of instability. However, in the case of an ACL rupture, the app algorithm had suggested to visit an emergency department, which would not have been suitable in this specific case with a chronic condition. Consequently, it can be assumed that the recommended visitation to a doctor – even though for the wrong primary diagnosis of bursitis - would have been beneficial for this case, likely eventually leading to a correct human-made diagnosis.

Various aspects must be critically discussed in the context of this report. One major limitation is that this case report only used one of various existing apps on the market, and the efficacy will likely vary between different algorithms [21]. In general, it has to be acknowledged that nowadays the purpose of AI-based chatbots cannot yet be seen as diagnosing complex clinical injuries or pathologies, instead is intended to give patients useful insight before getting a chance to meet or talk with a medical professional.

Another critical aspect, previously mentioned, is the potential dependence on the user’s understanding, as it has been shown that different users’ knowledge could lead to different results with an DDSS app [23]. It is therefore unclear whether the fictional patients would have given the same answers in the same way in real life achieving the same results presented here.

Critical aspects of current AI applications can also be suspected in the case of mild concussion or muscle pain. The fictitious clinical scenarios used in this study, involving these conditions, were all correctly diagnosed here. However, in a real medical setting, both scenarios can be fluid processes that - in extreme cases - could turn into a severe traumatic brain injury or a compartment syndrome. Thus, follow-ups should be provided by the app, if patients decide not to seek medical support.

This may pose legal problems: Is the manufacturer responsible, if the app does not recognize a pathology correctly? Patient confidence in an app can be high [12], even if the chatbot apps – like the one used in this report – indicate that they do not make any medical diagnoses or that qualified health care providers should be contacted regarding any medical issues [24]. However, this is still very vague in the current version, because especially in the case of concussions or compartment syndromes, hours could already bring dramatic changes. In addition to improving algorithms, a follow-up function by actively reminding the patient via the app (perhaps by push notification) would be a possible option - similar to a follow-up examination in a hospital setting.

In the context of DDSS apps, questions about how doctors should deal with false-positive findings will also be interesting - could this lead to an overtreatment for fear of legal consequences? On the other hand, what could the legal consequences be if physicians allowed themselves to be influenced by false negative app findings?

What is certain is that despite many human-made misdiagnoses, in up to 12 million cases or 5% of all adult patients in the USA [25], any serious AI errors will likely lead to similar media attention as seen today in a one-off accident with a semi-autonomously driving Tesla car, for example. One day, the use of AI may help to diagnose and even predict the occurrence of sport related injuries [26]. However, as of today, AI-based chatbots still appear to lag behind other algorithms using machine learning since natural language processing is still a complicated issue and most FDA approved AI-based medical technologies do not use it [27].

## Conclusions

DDSS apps certainly have a great potential in the analysis of patient complaints in the sports environment, especially when doctors or athletic trainers are not readily available during training or competition. However, many injuries will only be able to be assessed with certainty after a clinical examination and, if necessary, radiological diagnostics. Also, relevant therapy decisions will remain in the hands of physical therapists and athletic trainers, under the supervision of physicians. The app used did not underestimate the urgency of the constructed clinical pictures, although an improvement of the question-based diagnostic acuity still seems necessary in some areas. Potential can be seen in the use of the app as a triage-like pre-screening with a subsequent online video consultation where a more official medical assessment could be made, and if necessary, a physical doctor’s visit could be arranged. This and other points mentioned above should be the subject of further research.

## Supplementary Information

**Additional file 1: Supplement 1.** Generated by the App ADA for case 1 (“Tennis elbow”)

**Additional file 2: Supplement 2.** Generated by the App ADA for case 2 (“Ankle sprain”)

**Additional file 3: Supplement 3.** Generated by the App ADA for case 3 (“Delayed onset of muscle soreness”).

**Additional file 4: Supplement 4.** Generated by the App ADA for case 4 (“ACL rupture with chronic instability”).

**Additional file 5: Supplement 5.** Generated by the App ADA for case 5 (“Mild concussion”).

## Data Availability

The datasets used and analyzed during the current case report are available from the corresponding author on reasonable request.

## Abbreviations

ACL: Anterior crucial ligament
AI: Artificial intelligence
DDSS: Diagnostic decision support systems
dob: Date of birth
N/A: Not applicable
ROM: Range of motion
VAS: Visual analogue scale (for indicating pain intensity, increasing from 1 to 10)
y/o: Years old
