# Supplementary material for: Use of artificial intelligence in sports medicine: a report of 5 fictional cases
Source: BMC Sports Sci Med Rehabil. 2021 Feb 16;13:13. doi: 10.1186/s13102-021-00243-x (PMC7885566; doi:10.1186/s13102-021-00243-x)
Supplement: Supplementary file 1 — Additional file 1: Supplement 1. Generated by the App ADA for case 1 (“Tennis elbow”) [file 13102_2021_243_MOESM1_ESM.pdf]

Assessment Report

elbow pain

Michael M., Male, 1979

Reported symptoms

Symptoms reported as present

- Elbow pain
  - time since onset: one month to one year
  - activity: exacerbates
  - laterality: unilateral
  - intensity: moderate
- Elbow tenderness
  - laterality: unilateral
- Forearm pain
  - at motion: exacerbates
  - laterality: unilateral
  - intensity: moderate
- Regular physical activity
- Recent strenuous physical exercise
- Muscle tenderness of the arm

Symptoms reported as absent

- Tingling or numbness in the hand
- Lump under the skin on the elbow
- Lump under the skin on the lower arm
- Swollen elbow
- Reduced mobility of the elbow joint
- Arm or leg injury
- Muscle cramps in the arm and hand
- Tense arm muscles
- Bruise on the arm
- Reduced mobility of the fingers
- Reduced mobility of the wrist
- Warm elbow skin
- Reddened elbow skin
- Smoker
- Diabetes
- High blood pressure

Symptoms reported as unsure of

Possible causes

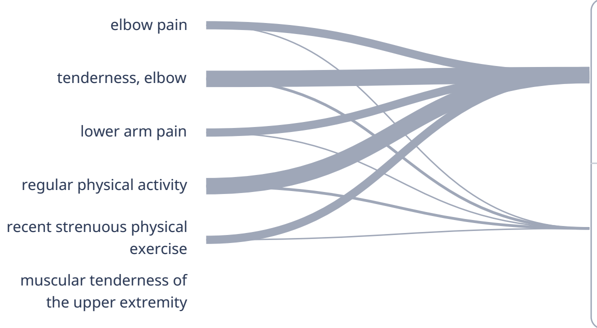

Tennis elbow
Can usually be managed at home

7 out of 10 people with these symptoms had this condition.

Golfer's elbow
Can usually be managed at home

2 out of 100 people with these symptoms had this condition.

Next Steps

People with symptoms similar to yours can usually manage their symptoms safely at home. You could also seek advice by visiting or contacting your local pharmacy. If your symptoms persist longer than expected, if they get worse, or if you notice new symptoms, you should consult a doctor for further assessment and advice.

1 Tennis elbow Can usually be managed at home

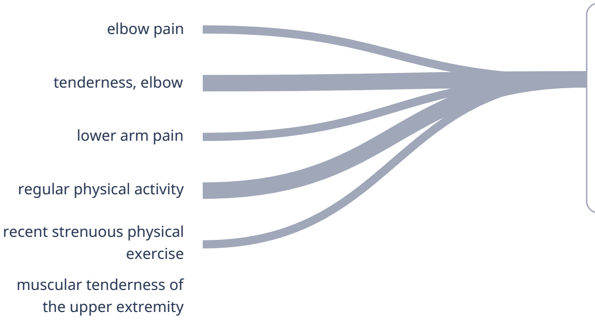

Tennis elbow

Can usually be managed at home

7 out of 10 people with these symptoms had this condition.

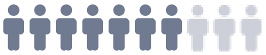

Description

Lateral epicondylitis, also known as tennis elbow, is a painful injury and inflammation due to overuse of tendons in the outer elbow. This injury usually occurs because of repetitive movements that occurred over a long period of time. The pain is commonly felt while lifting something, opening a door, raising hands, or making a fist. A doctor diagnoses the condition by assessing symptoms, examining the affected person, and sometimes imaging the elbow. Treatments consisting of rest, cryotherapy with ice packs, medication against pain and inflammation, and physiotherapy help relieve most people's symptoms.

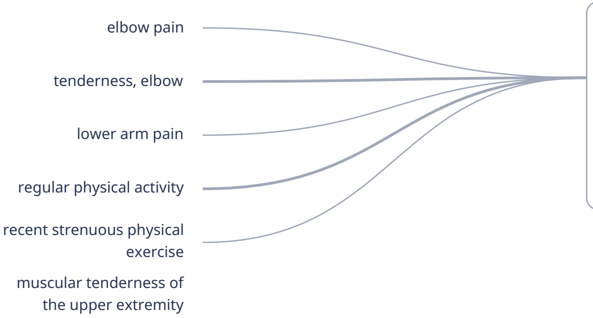

Golfer's elbow

Can usually be managed at home

2 out of 100 people with these symptoms had this condition.

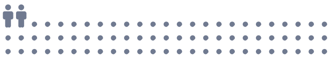

Description

Medial epicondylitis, or 'golfer's elbow' is a condition in which tendons on the inner side of the elbows become inflamed. This is usually due to overuse of the elbow. This often affects people who play golf or a sport which requires repeated throwing (such as baseball). Typical symptoms include elbow pain and hand weakness. Therapy for this condition includes taking painkillers, cooling the elbow with ice, and using an elbow brace to reduce further strain. The recovery outlook is good; the majority of affected people recover within a few months. Only a small percentage of people need further treatment, such as surgery.
