# Supplementary material for: Use of artificial intelligence in sports medicine: a report of 5 fictional cases
Source: BMC Sports Sci Med Rehabil. 2021 Feb 16;13:13. doi: 10.1186/s13102-021-00243-x (PMC7885566; doi:10.1186/s13102-021-00243-x)
Supplement: Supplementary file 2 — Additional file 2: Supplement 2. Generated by the App ADA for case 2 (“Ankle sprain”) [file 13102_2021_243_MOESM2_ESM.pdf]

Assessment Report

ankle pain

Sarah s., Female, 1993

Reported symptoms

Symptoms reported as present

- Ankle pain
  - time since onset: less than one day
  - activity: exacerbates
  - laterality: unilateral
  - intensity: severe
- Arm or leg injury
- Ankle swelling
  - laterality: unilateral
- Ankle tenderness
  - laterality: unilateral
- Reduced mobility of ankle joint
- Swelling on the outer side of the ankle
- Inability to bear weight
- Lateral ankle tenderness
- Recent strenuous physical exercise
- Warm skin at ankle

Symptoms reported as absent

- Misshapen ankle
- Bruise on the ankle
- Tenderness on the inner side of the ankle
- Foot pain
- Swelling of the heel
- Swelling on the inner side of the ankle
- Muscle weakness in leg
- Lower leg pain
- Pale leg
- Bruise on the foot
- Swelling of the top of the foot
- Reddened foot skin
- Smoker
- Pregnant
- Diabetes
- High blood pressure

Symptoms reported as unsure of

- Ankle feels unstable

Possible causes

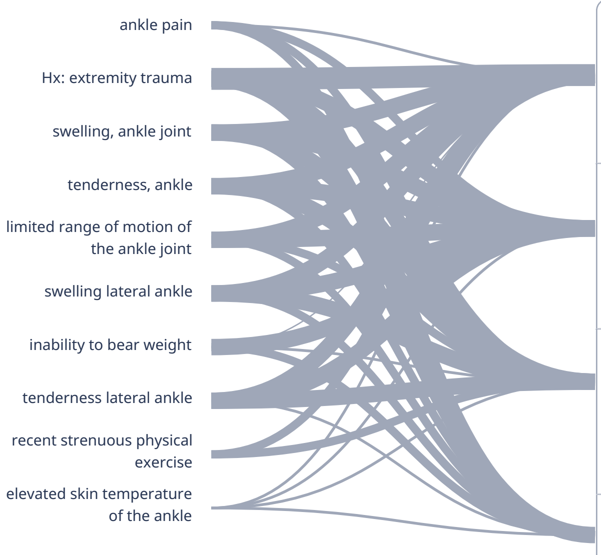

Sprained ankle without ligament rupture

Seek emergency care

3 out of 10 people with these symptoms had this condition.

Lateral malleolus fracture

Seek emergency care

2 out of 10 people with these symptoms had this condition.

Lateral ligament rupture of the ankle

Seek emergency care

2 out of 10 people with these symptoms had this condition.

Ankle fracture, not further specified

Seek emergency care

1 out of 10 people with these symptoms had this condition.

Next Steps

People with symptoms similar to yours may require emergency care. If you think this is an emergency you should go to an emergency department without delay.

1 **Sprained ankle without ligament rupture** Seek emergency care

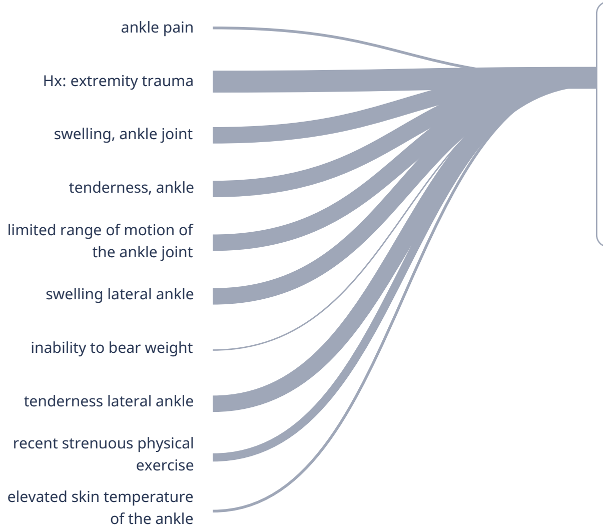

Sprained ankle without ligament rupture

Seek emergency care

3 out of 10 people with these symptoms had this condition.

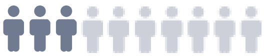

Description

A sprained ankle is a common condition caused by overstretching or tearing the ankle ligaments, the fibers which connect bones. Athletes are especially at risk for developing this condition, usually due to quick movements of the feet. Symptoms in the ankle include pain, stiffness, and swelling. A diagnosis is usually based on a physical examination and imaging tests such as X-rays. Treatment can involve rest, pain relievers, heat and ice packs, and physiotherapy. Certain measures can help with prevention, such as warming-up before exercise and wearing adequately supportive shoes.

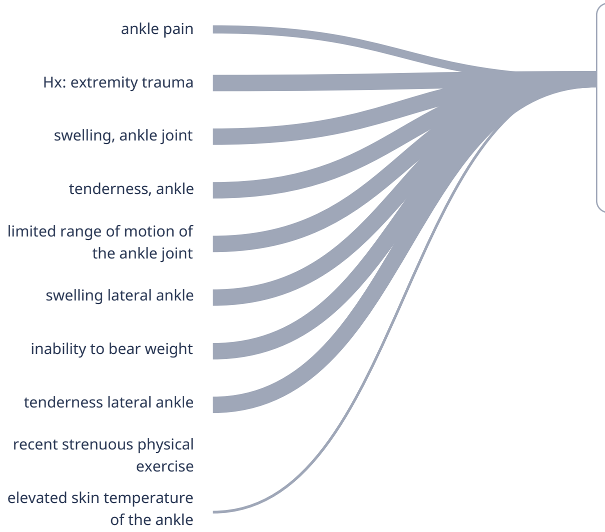

Lateral malleolus fracture

Seek emergency care

2 out of 10 people with these symptoms had this condition.

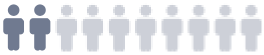

Description

A lateral malleolus fracture is the medical term for a break in the outer ankle bone. This is a common injury which often occurs after twisting or rolling the ankle. It can occur in people of all ages, but is slightly more common in older people. Symptoms of this injury include ankle swelling, pain when pressing the outer ankle bone, and difficulty bearing weight on the ankle. The diagnosis is usually confirmed with an X-ray or CT scan of the ankle. In many instances, the break is treated by using a cast to keep the ankle immobile, although larger injuries may be treated with surgical repair of the bones. Uncomplicated breaks usually heal well, though it may be 6 or 10 weeks before weight can be put on the foot or ankle.

3 Lateral ligament rupture of the ankle Seek emergency care

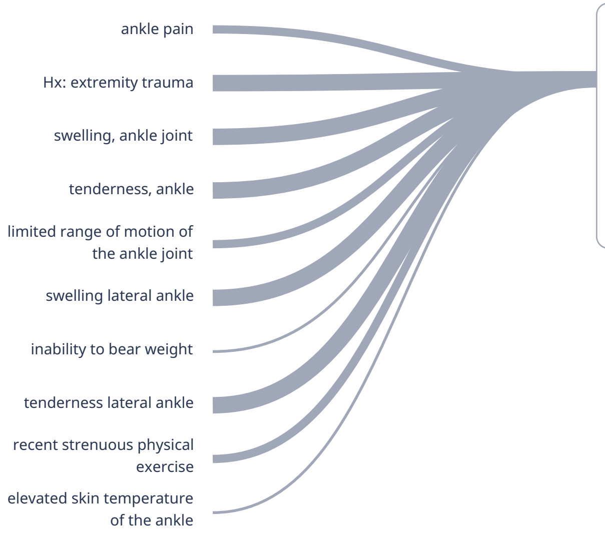

Lateral ligament rupture of the ankle

Seek emergency care

2 out of 10 people with these symptoms had this condition.

Description

Lateral ligament rupture is the medical term for a tear of one of the ligaments that give support to the ankle. It is a severe form of sprained ankle that occurs after movements like rolling or twisting the ankle, usually while doing sports. The symptoms of this condition often include moderate to severe pain when putting weight on the injured foot, limping, and a hematoma (blue discoloration) on the ankle. The diagnosis is based on the symptoms and a physical examination. An X-ray of the ankle can be done to exclude bone fractures. Treatment usually consists of cooling, rest, and elevation of the injured foot, and taking pain medication. An ankle support might also help. Most people with a lateral ligament rupture recover very well. It is important to follow a good rehabilitation program to prevent new injuries of the ankle.

4 Ankle fracture, not further specified

Seek emergency care

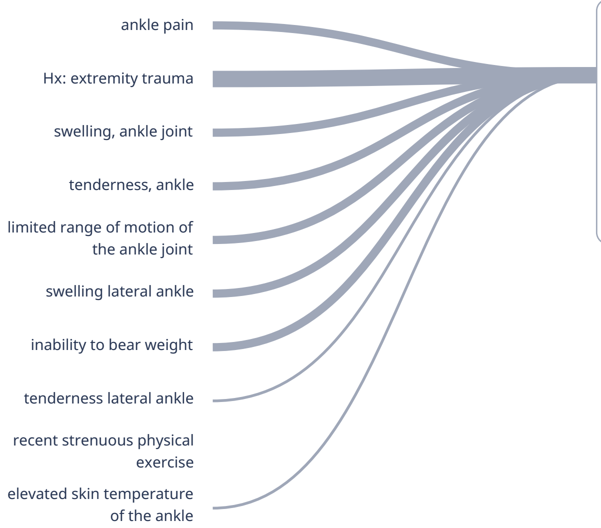

Ankle fracture, not further specified

Seek emergency care

1 out of 10 people with these symptoms had this condition.

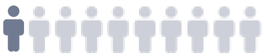

Description

An ankle fracture is the medical term for a break in one or several bones of the ankle. This is a common bone fracture that may occur due to twisting the ankle, falling, or having an accident. Symptoms include pain, swelling, bruising, or a faulty position of the ankle and foot. Immediately after an ankle injury, it is generally advisable to keep weight off the foot and apply basic first aid. A doctor diagnoses the condition by assessing the symptoms, examining the ankle, and possibly ordering imaging tests such as an X-ray or MRI. Treatment depends on the type and severity of the ankle fracture and may consist of pain medication, immobilization with a cast, or surgery. A full recovery usually takes around 2 months.
