# Supplementary material for: Use of artificial intelligence in sports medicine: a report of 5 fictional cases
Source: BMC Sports Sci Med Rehabil. 2021 Feb 16;13:13. doi: 10.1186/s13102-021-00243-x (PMC7885566; doi:10.1186/s13102-021-00243-x)
Supplement: Supplementary file 3 — Additional file 3: Supplement 3. Generated by the App ADA for case 3 (“Delayed onset of muscle soreness”). [file 13102_2021_243_MOESM3_ESM.pdf]

Assessment Report

pain in the leg

Peter P., Male, 2001

Reported symptoms

Symptoms reported as present

- Pain in the leg
  - time since onset: one day to one week
  - at motion: exacerbates
  - laterality: bilateral
  - intensity: severe
- Muscular tenderness of the leg
- Tender thigh
- Thigh pain
- Limping
- Pain in the front or outer side of the thigh
- Recent strenuous physical exercise
- Leg feels heavy
- Buttock pain

Symptoms reported as absent

- Bruise on the thigh
- Swelling of the thigh
- Inability to bear weight
- Reduced mobility of the knee joint
- Arm or leg injury
- Calf pain
- Pain in the back of the thigh
- Lump under the skin on the thigh
- Muscle cramps in leg
- Smoker
- Diabetes
- High blood pressure

Symptoms reported as unsure of

Possible causes

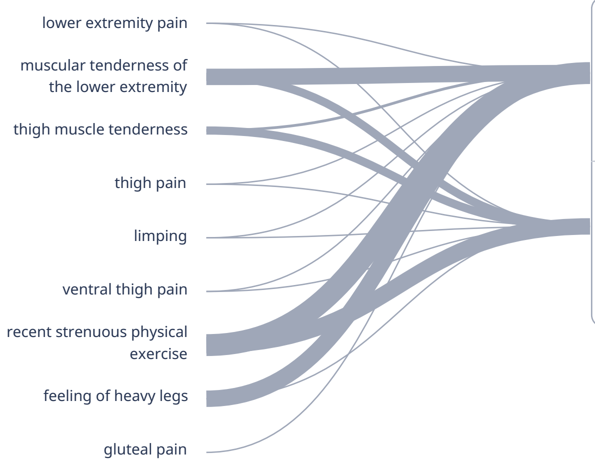

Delayed-onset muscle soreness of the lower extremity

Can usually be managed at home

5 out of 10 people with these symptoms had this condition.

Quadriceps strain

Can usually be managed at home

2 out of 10 people with these symptoms had this condition.

Next Steps

People with symptoms similar to yours can usually manage their symptoms safely at home. You could also seek advice by visiting or contacting your local pharmacy. If your symptoms persist longer than expected, if they get worse, or if you notice new symptoms, you should consult a doctor for further assessment and advice.

1 Delayed-onset muscle soreness of the lower extremity

Can usually be managed at home

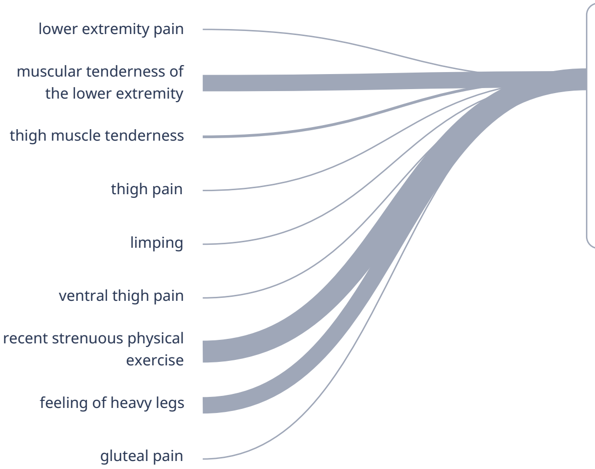

Delayed-onset muscle soreness of the lower extremity

Can usually be managed at home

5 out of 10 people with these symptoms had this condition.

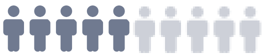

Description

Delayed-onset muscle soreness is muscle pain and stiffness which occurs after strenuous exercise. The pain is at its worst 24 to 72 hours after the exercise session, and is typically dull, aching, and worse when using the muscle. The muscle also often feels stiff, and sore when pressed. These symptoms are caused by microscopic muscle damage which occurs during exercise, especially if the workout involves muscle groups that are not often used, or if it is longer or more intense than usual. The symptoms usually get better without specific treatment within one week. Gentle massage or warm baths can help to manage the pain and stiffness.

2 Quadriceps strain
Can usually be managed at home

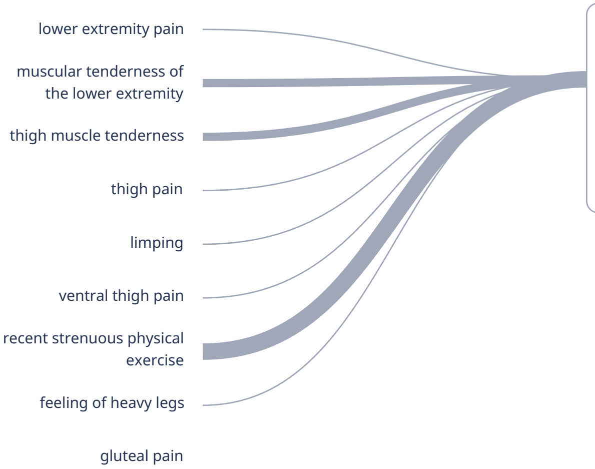

Quadriceps strain
Can usually be managed at home

2 out of 10 people with these symptoms had this condition.

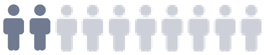

Description

A quadriceps strain is a condition in which there is overstretching or tearing of the quadriceps muscles (the 'quads'). These are the large muscles at the front of the thigh. This injury most commonly occurs when the muscle is used in a powerful movement, such as when jumping, sprinting or kicking. People with a quadriceps strain have pain in the thigh which may be worse with movement of the knee and hip. There may be swelling and bruising in the area of the injury. This condition may be diagnosed based on the symptoms and examination, but an ultrasound or another scan may be needed to diagnose a large injury to the muscle. A quadriceps strain is treated by resting the muscle, applying a cold compress to the area, and with simple pain-relief. Recovery time depends on the size of the injury, and may take from 2 weeks to several months.
