# Supplementary material for: Use of artificial intelligence in sports medicine: a report of 5 fictional cases
Source: BMC Sports Sci Med Rehabil. 2021 Feb 16;13:13. doi: 10.1186/s13102-021-00243-x (PMC7885566; doi:10.1186/s13102-021-00243-x)
Supplement: Supplementary file 4 — Additional file 4: Supplement 4. Generated by the App ADA for case 4 (“ACL rupture with chronic instability”). [file 13102_2021_243_MOESM4_ESM.pdf]

Assessment Report

swollen knee

Thomas T., Male, 1994

Reported symptoms

Symptoms reported as present

- Swollen knee
  - time since onset: one month to one year
- Knee pain
  - time since onset: one month to one year
  - activity: exacerbates
  - laterality: unilateral
  - intensity: moderate
- Regular physical activity
- Recent strenuous physical exercise
- Arm or leg injury
- Knee feels unstable
- Smoker

Symptoms reported as absent

- Tender knee
- Lump under the skin behind the knee
- Pain in the bony area below the knee
- Knee pain, outer side
- Pain around the kneecap
- Morning stiffness
- Warm knee skin
- Shin pain
- Limping
- Difficulty walking
- Knee pain, inner side
- Lump under the skin over a joint
- Reduced mobility of the knee joint
- Reddened knee skin
- Inability to bear weight
- Bruise on the knee
- Calf pain
- Pain in the back of the knee
- Swelling of the leg
- Diabetes
- High blood pressure

Symptoms reported as unsure of

Possible causes

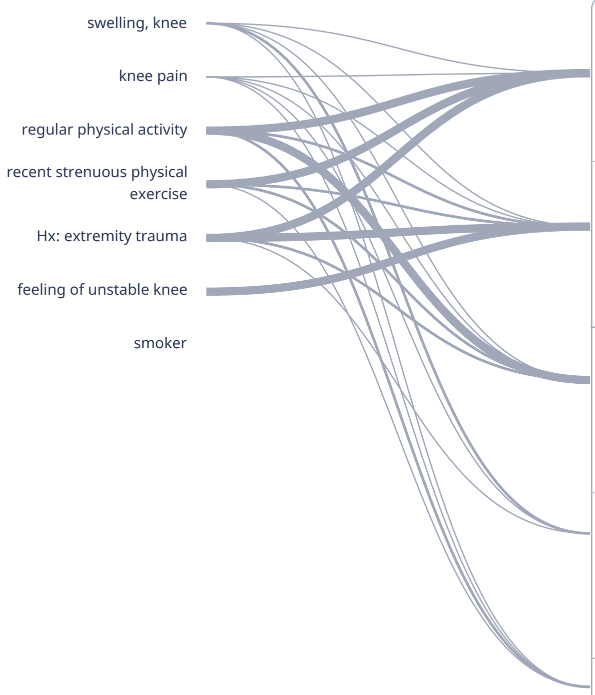

Knee bursitis
Seek medical advice

3 out of 10 people with these symptoms had this condition.

Anterior cruciate ligament injury
Seek emergency care

1 out of 10 people with these symptoms had this condition.

Patellar tendinitis
Can usually be managed at home

8 out of 100 people with these symptoms had this condition.

Popliteal cyst
Seek medical advice

7 out of 100 people with these symptoms had this condition.

Iliotibial band syndrome
Can usually be managed at home

4 out of 100 people with these symptoms had this condition.

Next Steps

People with symptoms similar to yours may require emergency care. If you think this is an emergency you should go to an emergency department without delay.

1 # Knee bursitis
Seek medical advice

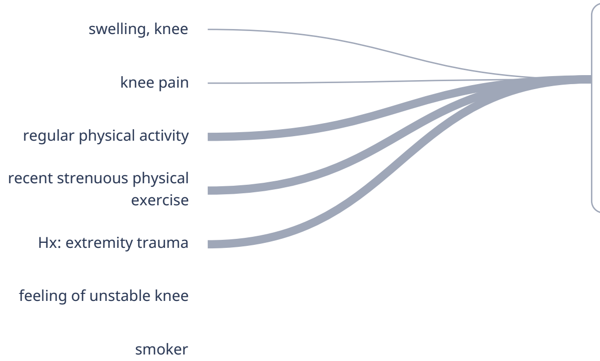

Knee bursitis

Seek medical advice

3 out of 10 people with these symptoms had this condition.

Description

Knee bursitis is a painful inflammation of one of the small fluid-filled sacs (the bursa or bursae) which is located around the knee joint. Bursae can be found around most joints, and protect the bones and tendons from pressure and friction. Repetitive movements (such as lifting), holding certain positions (leaning, kneeling), or a direct blow to the knee can irritate the bursae. Symptoms include pain when moving the knee, swelling, redness, and stiffness. The diagnosis can usually be made based on examination of the knee, but in some cases, further tests may be needed to confirm the diagnosis. Treatment includes rest, ice packs, simple pain-relief and anti-inflammatory medications, and physiotherapy. In severe cases, surgical drainage or removal of a bursa may be useful. Most people respond well to treatment and have no lasting problems, although bursitis can reoccur if the bursa becomes irritated again.

2 Anterior cruciate ligament injury Seek emergency care

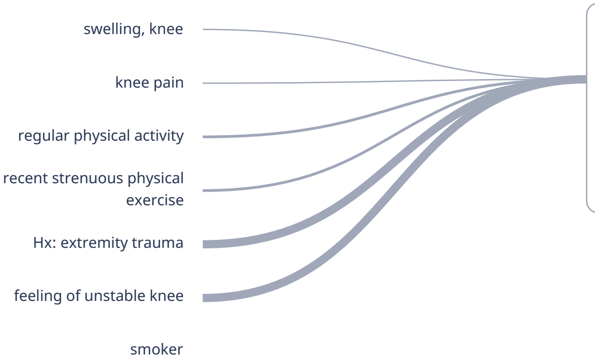

Anterior cruciate ligament injury

Seek emergency care

1 out of 10 people with these symptoms had this condition.

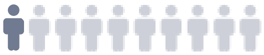

Description

Located at the knee joint, the anterior cruciate ligament (ACL) stabilizes the knee by preventing the lower leg from moving too far forward. Injury to this ligament is often sports-related. An ACL injury can cause swelling, pain, instability, and a popping sound inside the knee when the ligament tears. The injury is usually diagnosed by physical examination and medical imaging studies. Treatment consists of rest, cooling with ice, compression and elevation of the affected knee. In some cases, surgery might be needed. Depending on the severity of the injury, recovery lengths vary.

Patellar tendinitis

Can usually be managed at home

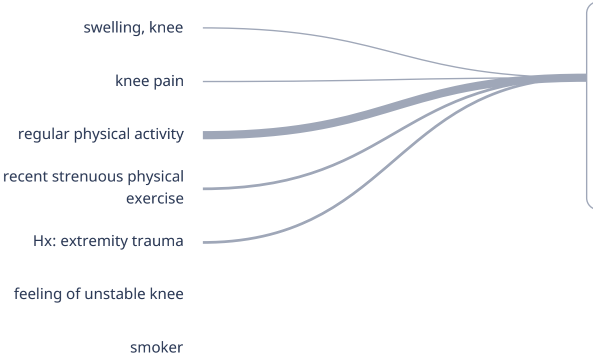

Patellar tendinitis

Can usually be managed at home

8 out of 100 people with these symptoms had this condition.

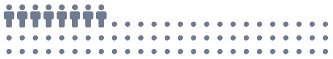

Description

Patellar tendinitis, also known as jumper's knee, is a condition in which the patella tendon becomes painful and swollen due to overuse. The patella tendon joins the knee cap to the shin bone. This condition most commonly occurs in athletes, especially those who participate in sports that involve frequent jumping. The most common symptoms are pain with bending the knee (squatting, sitting, etc.), and a tender swelling below the knee cap. Treatment consists of resting, cooling the area with ice, and simple pain relief (paracetamol or ibuprofen as needed). In most cases, the symptoms will improve of a number of days, however, the symptoms may recur. Physiotherapy may be useful for active people in order to stretch and strengthen the muscles around the knee and avoid future episodes of patellar tendinitis.

- swelling, knee
- knee pain
- regular physical activity
- recent strenuous physical exercise
- Hx: extremity trauma
- feeling of unstable knee
- smoker

Popliteal cyst

Seek medical advice

7 out of 100 people with these symptoms had this condition.

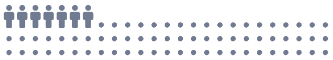

Description

A popliteal cyst, or Baker's cyst, is a fluid-filled sac (cyst which forms at the back of the knee). In adults, it is often associated with osteoarthritis or knee cartilage damage. Symptoms can include pain, swelling, and limited movement of the knee. The cyst can be diagnosed with a physical examination and ultrasound. These cysts usually do not need treatment and may disappear without treatment, or may burst, causing swelling of the calf. Steps that may help to relieve symptoms include: resting the leg; applying a cold pack; elevating the leg; and simple pain-relief (paracetamol or ibuprofen). Most people recover well from a Baker's cyst, though if the underlying cause is not treated, the cyst may reoccur.

5 **Iliotibial band syndrome** Can usually be managed at home

swelling, knee

knee pain

regular physical activity

recent strenuous physical exercise

Hx: extremity trauma

feeling of unstable knee

smoker

Iliotibial band syndrome

Can usually be managed at home

4 out of 100 people with these symptoms had this condition.

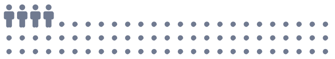

Description

Iliotibial band syndrome, also known as runner's knee, is a painful injury due to overuse of the connective tissues on the outer side of the knee. This usually affects long-distance runners or bicyclists with poor mechanical balance in the back, hips, and knees. The pain can progress to become a disabling factor while even walking or climbing steps. Sometimes there is a audible snapping sound or notable swelling at the knee. The diagnosis is based on symptoms and a physical examination. Treatment may consist of rest, cryotherapy with ice packs, medication against pain and inflammation, physiotherapy, and exercises to stretch and strengthen affected knee muscles.
