# Supplementary material for: Use of artificial intelligence in sports medicine: a report of 5 fictional cases
Source: BMC Sports Sci Med Rehabil. 2021 Feb 16;13:13. doi: 10.1186/s13102-021-00243-x (PMC7885566; doi:10.1186/s13102-021-00243-x)
Supplement: Supplementary file 5 — Additional file 5: Supplement 5. Generated by the App ADA for case 5 (“Mild concussion”). [file 13102_2021_243_MOESM5_ESM.pdf]

ada

Assessment Report

headache

Marc C., Male, 1997

Reported symptoms

Symptoms reported as present

- Headache
  - pounding headache: no
  - bending forward: exacerbates
  - time since onset: less than one day
  - intensity: severe
- Dizziness
  - standing up: exacerbates
  - type of dizziness: lightheadedness
  - time since onset: less than one day
- Head or neck injury
- Recent sun or heat exposure
- Scalp sore to touch
- Nausea
  - intensity: moderate
- Memory loss
- Balance problems
- Smoker

Symptoms reported as absent

- Loss of consciousness
- Sudden confusion
- Discharge from ear
- Reduced mobility of neck
- Bruise on the scalp
- Neck pain
- Lump under the skin on the scalp
- Recent decrease in alcohol intake
- Disorientation
- Pain around the eye
- Jerking movements of the whole body
- Pain behind the eye
- Reddened facial skin
- Runny nose
- Vomiting
- Facial swelling
- Double vision
- Diabetes
- High blood pressure

Symptoms reported as unsure of

Possible causes

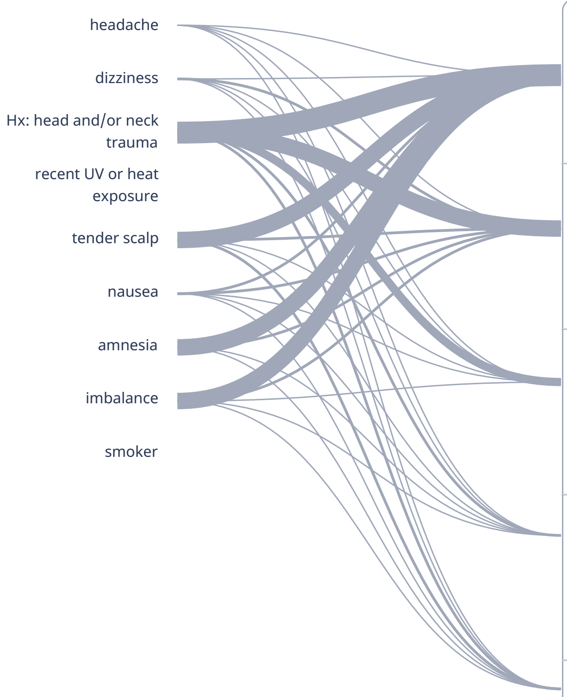

Concussion
Seek medical advice

5 out of 10 people with these symptoms had this condition.

Acute subdural hematoma
Seek emergency care

1 out of 10 people with these symptoms had this condition.

Whiplash
Seek medical advice

3 out of 100 people with these symptoms had this condition.

Acute intracranial epidural hematoma
Seek emergency care

2 out of 100 people with these symptoms had this condition.

Skull fracture
Seek emergency care

1 out of 100 people with these symptoms had this condition.

Next Steps

People with symptoms similar to yours may require emergency care. If you think this is an emergency the safest thing to do is call an ambulance.

1 Concussion Seek medical advice

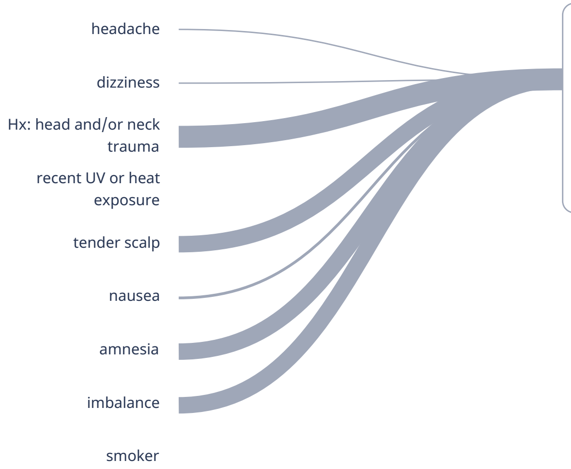

Concussion

Seek medical advice

5 out of 10 people with these symptoms had this condition.

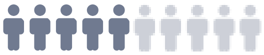

Description

A concussion is a minor and temporary injury to the brain. Concussion can cause headache, confusion, tiredness, mood changes and nausea. If symptoms occur after a blow to the head, one should be assessed by a doctor and should be observed carefully for 24 hours for signs of a more severe brain injury. People who are unconscious for more than 5 minutes, who have severe memory loss, who have a discharge from their nose or black eyes without injury need urgent review by a doctor. People with a concussion should rest until their symptoms settle. Concussion usually gets better within 1 to 2 days, but some people experience on-going symptoms for up to 6 months.

2

Acute subdural hematoma

Seek emergency care

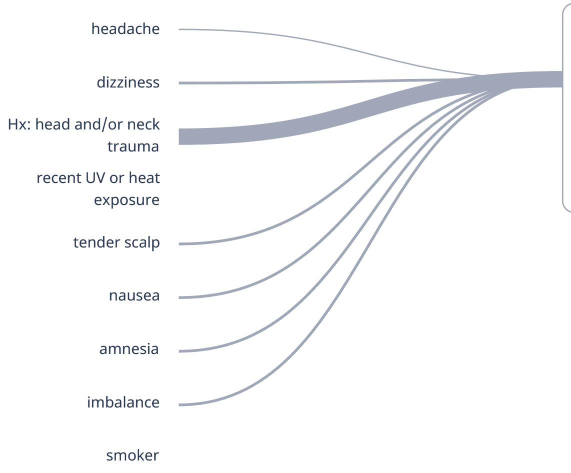

Acute subdural hematoma

Seek emergency care

1 out of 10 people with these symptoms had this condition.

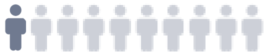

Description

An acute subdural hematoma is when blood collects between the brain and the dura mater, its outer layer. This emergency condition is usually associated with a sudden and severe head injury. Symptoms appear right away and may include headache, confusion, nausea and vomiting, or in severe cases, unconsciousness or coma. Diagnosis involves medical imaging such as computed tomography (CT) or magnetic resonance imaging (MRI). This condition demands prompt and adequate intervention. Treatment depends on the severity of the hematoma but very often involves brain surgery.

3 **Whiplash** Seek medical advice

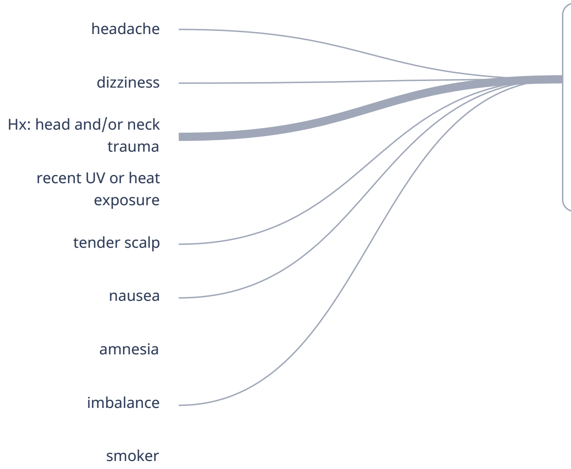

Whiplash

Seek medical advice

3 out of 100 people with these symptoms had this condition.

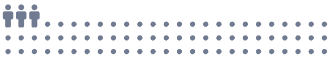

Description

Whiplash-associated disorder (WAD), or a whiplash injury, is an orthopedic condition due to distortion of the neck from a sudden jolting motion of the head. The most common cause is a motor vehicle accident (getting hit by a vehicle from behind), when the head suddenly moves backwards and forwards relative to the body. Women have a higher risk for WAD because of weaker neck muscles. Typical symptoms are neck pain or stiffness after the accident, headache, dizziness, vision problems, and numbness or weakness of the shoulders and arms. Treatment can consist of painkillers and physical therapy and in severe injuries, surgery. Psychological support is advised to patients with the risk of experiencing chronic pain.

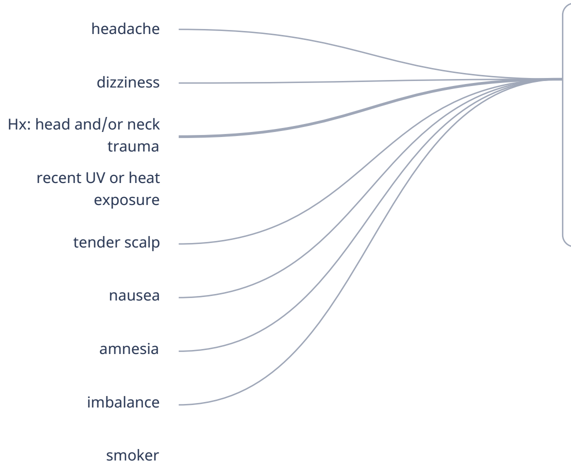

Acute intracranial epidural hematoma

Seek emergency care

2 out of 100 people with these symptoms had this condition.

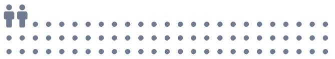

Description

An intracranial epidural hematoma is a condition in which there is bleeding in the space between the outer layer of the brain and the skull bone. This is an emergency and needs urgent review by a doctor. Head injuries are the most common cause. Symptoms may include headache, confusion and loss of consciousness. The diagnosis is confirmed with a CT scan of the head. Treatment depends on the severity of the bleeding, but often involves surgery.

5 Skull fracture Seek emergency care

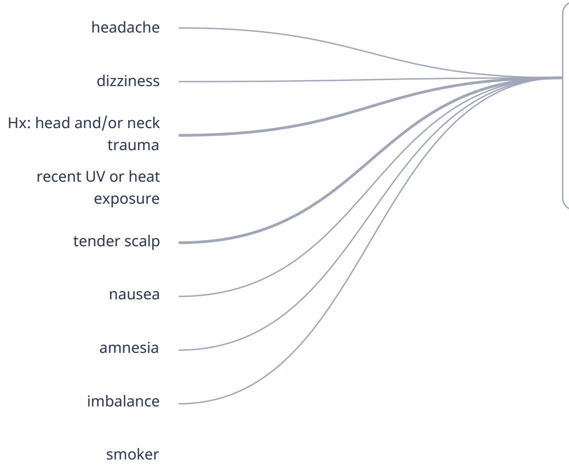

Skull fracture

Seek emergency care

1 out of 100 people with these symptoms had this condition.

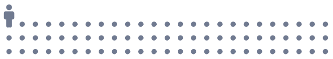

Description

Skull fractures are breaks in the cranial (head) bones usually following a physical trauma, like a forceful impact or car accident. An ambulance should be immediately called if the affected person suffers even a partial loss of consciousness or signs of neurological disturbance, for instance confusion, loss of smell, vision, or coordination. Many skull fractures require surgical intervention, in some cases urgently. Facial bone fractures are the most common locations, particularly on the jaw and cheek bones. Diagnosis depends on the how the injury occurred, any complaints, a physical examination, and imaging tests. The recovery outlook varies greatly and depends on whether brain damage was sustained. Protective helmets can reduce the risk of skull fractures, for example in motorcyclists, construction workers, and ice hockey players.
